# Cancer Is A Survival Process under Persistent Microenvironmental and Cellular Stresses

**DOI:** 10.1016/j.gpb.2022.03.002

**Published:** 2022-06-18

**Authors:** Renbo Tan, Yi Zhou, Zheng An, Ying Xu

**Affiliations:** 1 Cancer Systems Biology Center, China-Japan Union Hospital of Jilin University, Changchun 130000, China; 2 College of Computer Science and Technology, Jilin University, Changchun 130000, China; 3 Department of Biochemistry and Molecular Biology, and Institute of Bioinformatics, University of Georgia, Athens, GA 30602, USA

What defines cancer? Numerous factors have been found to be associated with cancer onset and development. Rudolf Virchow suggested some 150 years ago that cancer was causally linked to persistent irritation. Later studies have revealed that overexposure to radiation, persistent microbial infection, genetic mutations, and chronic (sterile) inflammation could all potentially lead to cancer, and the same has been observed about intermittent hypoxia, iron overload, and intake of certain “carcinogens”. In parallel, considerable information has been collected regarding animals that never or rarely develop cancer, such as blind mole rats and whales. Yet no general frameworks have been published that can functionally link potential drivers to the formation and progression of cancer in a predictable manner, which is consistent with the aforementioned and other epidemiological data. Here, we outline one such framework.

## Study of cancer in an evolutionary framework

The cancer-defining question has been actively studied since at least as early as Rudolf Virchow’s proposal [1]. Otto Warburg observed in 1927 that cancer cells rely more on the fermentation pathway for energy generation in addition to the respiration pathway, which is used solely by normal non-proliferating cells for energy production [2], and he later suggested this switch as a fundamental cause of cancer in general [3]. Since late 1970s, cancer researchers have gradually accepted the hypothesis that cancer is the result of genomic mutations after the publication of two seminal papers: the study on proto-oncogenes and the driving roles of their mutations in cancer by Stehelin et al. [4] and the work on tumor suppressor genes and their guarding roles against cancer by Knudson AG [5], both in 1970s. While this view of cancer remains mainstream thinking, it has been challenged in the past decade [6], [7]. Reasons include that (1) no general and testable framework has been established that can causally link mutations to a wide range of cancerous behaviors, such as the extensively reprogrammed metabolisms (RMs) that are conserved across cancer types [8], [9] and the hallmarks of cancer [10]; and (2) treatments developed based on the mutation-centric view of cancer have yet to make quantum leaps in lowering cancer death rates because of their seemingly intrinsic drug resistance issues.

Research in microbiology and plant biology has established an effective framework for studying organismic evolution. Briefly, it is stresses induced by organismic competition or environmental changes that lead to increased production of reactive oxygen species, resulting in genetic mutations and epigenomic changes, termed stress-induced mutagenesis [11]. Some of the mutations and/or epigenomic alterations are selected to enable the stressed cells to adapt and survive. This framework is consistent with Darwin’s theory of evolution and has been applied successfully to study how organisms adapt to a wide range of stressors [12], [13], including cancer [11]. Our own study on how *Escherichia coli* adapts to alcohol-induced stress has revealed that different *E. coli* cells may adapt to and survive the stress via the selection of distinct mutations [14], indicating that different mutations could be selected by individual cells to survive the same stress. This also suggests that genetic heterogeneity may not be necessarily a puzzling issue but instead a natural result of different cells having selected distinct mutations to overcome a common stressor. In contrast to the mainstream view of cancer research, mutations here are not the cause but a facilitator of organismic evolution, which is also the result of stresses.

Is it possible that cancer evolution follows a similar process? If yes, what are the stressors that drive the onset and progression of cancer? Knowing that different cancer types share considerably similar behaviors, such as the cancer hallmarks and the extensive and conserved RMs, we hypothesize that different cancers may be driven by the same (to-be-identified) molecular-level stressors.

It has been established that cancer tissue cells consume considerably more glucose than normal matching cells, generally up to 20–30 folds more [15], which is the basis for cancer detection using positron emission tomography/computed tomography (PET/CT) scan. In addition, these cells are under substantial stress(es) as reflected by their high levels of mutations, substantially up-regulated proteasomes for degradation of damaged proteins, and highly increased endoplasmic reticulum stress. Furthermore, more than 95% of the cancer cells die soon after their creation [16]. All these reveal that the cost for cancerous development is very high, hence suggesting that the cost for the alternative, *i.e.*, not selecting the trajectory of a cancerous evolution, must be even higher. We hypothesize that the cancer-defining stress(es) will lead to 100% cell death unless the cells divide persistently. A key difference between our view *vs.* the genetic-centric view is that cancerous cell division is a “must do” act for survival rather than being instructed by mutated genetic codes.

## RMs and cytosolic pH

Numerous metabolisms are known to be altered in a conserved manner across multiple cancer types. The Warburg effect is the first observed RM. The following are some other examples. Cancer relies more on *de novo* synthesis to produce nucleotides for DNA synthesis rather than through uptake of nucleosides from circulation followed by phosphorylation, which is more energetically favorable, a discovery made in 1970s [17]. Our own research has further revealed that more malignant cancers rely more on *de novo* synthesis than uptake [9]. Another class of conserved RMs is exemplified by the simultaneous synthesis and degradation of triglycerides. Other examples of RMs include (1) inhibition/repression of the urea cycle for releasing ammonia, the waste of amino acid metabolism [18], and (2) altered branched-chain amino acid metabolisms [19] across multiple cancer types.

Proposals have been made regarding the possible reasons for these and other commonly observed RMs in cancer, generally following the idea that these RMs enable the activation of oncogenes and/or production of oncometabolites [20], [21]. We have previously studied ∼ 50 such RMs (see Table S1) in over 7000 tissues of 14 cancer types in The Cancer Genome Atlas (TCGA) database and discovered that each of the RMs produces more or consumes fewer protons compared to its original metabolism [9]. These 14 cancer types are bladder urothelial carcinoma (BLCA), breast invasive carcinoma (BRCA), colon adenocarcinoma (COAD), esophageal carcinoma (ESCA), head and neck squamous cell carcinoma (HNSC), kidney chromophobe (KICH), kidney renal clear cell carcinoma (KIRC), kidney renal papillary cell carcinoma (KIRP), liver hepatocellular carcinoma (LIHC), lung adenocarcinoma (LUAD), lung squamous cell carcinoma (LUSC), prostate adenocarcinoma (PRAD), stomach adenocarcinoma (STAD), and thyroid carcinoma (THCA). It is noteworthy that cancer cells in general, have elevated cytosolic pH *vs.* their matching normal cells, approximately 7.2–7.4 *vs.* 6.8–7.0 [22], [23]. These, along with the observation that cancer tissue cells tend to increase proton-absorbing pumps and decrease proton-releasing pumps [24], lead naturally to the following hypothesis: there must be unknown metabolic processes that continuously produce alkaline molecules across many, possibly all cancer types.

## Chronic inflammation, iron overload, and Fenton reaction

Our search for the unknown alkalinizing processes has led us to focus on chronic inflammation, which has been accepted as being causally linked to cancer onset and development in general. A key characteristic of an inflammatory site is the increased concentrations of H_2_O_2_ and ĊO_2_^−^ (superoxide), released by neutrophil and microphage cells. Once such concentrations are beyond a certain level, red blood cells in the vicinity may die due to oxidative damages, hence leading to local accumulation of iron, which is carried by the blood cells. In addition, human epithelial cells are programmed to sequester nearby iron in the extracellular space when perceiving microbial invasion [25], which is the case for an inflammatory site. When H_2_O_2_ and ĊO_2_^−^ meet with Fe^2+^ at sufficiently high concentrations, Fenton reaction, a reaction with no need of enzymatic catalysis, will take place:

(1) Fe^2+^ + H_2_O_2_ → Fe^3+^ + OH^−^ + ĊOH

Multiple studies have reported that cancer tissue cells harbor Fenton reactions [26], [27]. Based on statistical analyses and modeling of transcriptomic data in TCGA, we have demonstrated that the majority, possibly all of the cancer tissue cells harbor Fenton reactions in their cytosol, mitochondria, extracellular matrix, and cell surface, each serving a distinct role in cancer development [28]. The reaction continues as long as there are reducing molecules nearby that can convert Fe^3+^ back to Fe^2+^. Our analyses suggest that cancer tissue cells generally use ĊO_2_^−^ as the reducing molecule, giving rise to repeated Fenton reactions, also called the Haber–Weiss reaction:

(2) H_2_O_2_ + ĊO_2_^−^ → OH^−^ + ĊOH + O_2_

with Fe^2+^ serving as a catalyst. While Fenton reactions may produce various other products, OH^−^ and ĊOH represent the dominant products [29].

Virtually all published studies on cancer-related Fenton reactions focus on the damaging effect of the produced hydroxyl radical (ĊOH) [30], while we have been focusing on the impact of the produced OH^−^. We have shown that the rate of OH^−^ production by cytosolic Fenton reaction, estimated based on relevant gene expression data of cancer tissues, will overwhelm the intracellular pH buffer quickly [28] and drive the pH up, hence casting major stress on the affected cells since all cells must stay within a narrow range of pH to be viable. Note that this stress cannot be easily resolved by pumping in protons or out hydroxides in a sustained manner since both are electrically charged, otherwise, it will result in persistent violation of the cellular electroneutrality, a fundamental property that cells must maintain to be viable [31]. We have shown that the predicted rate of cytosolic Fenton reaction strongly correlates with the combined rate of proton production of the observed RMs in cancer tissues [9]. This, along with additional evidence, gives rise to our prediction: RMs observed in each cancer are induced to produce protons at a comparable rate of OH^−^ production in cells harboring persistent cytosolic Fenton reactions [9].

## Cell division for survival, cellular transformation, and other cancerous behaviors

Among all the RMs, a few are highly conserved across many, possibly all cancer types in TCGA. *De novo* synthesis of nucleotides is one, possibly because it is a powerful proton producer as, for example, the synthesis of a purine produces 8–9 net protons [9]. While nucleotide synthesis helps to keep the intracellular pH stable, the synthesized nucleotides, at a rate dictated by the level of cytosolic Fenton reaction, must be consumed or released from the cells in a timely manner to sustain this proton-producing, hence life-saving process. However, this is not easy as each nucleotide carries a negative charge, and releasing them in a sustained manner will violate cellular electroneutrality. Here we ask: is it possible that Fenton reaction-affected cells may utilize cell division as a sustainable way to remove nucleotides at rates compatible with the rates of OH^−^ production by persistent Fenton reactions?

Unicellular organisms like *E. coli* are known to keep eating as long as the food is available [32]. The nutrient will be first converted to adenosine triphosphates (ATPs) till the cellular ATP concentration reaches a certain threshold, and then the cells switch to nucleotide synthesis. In such cells, the concentration of nucleotides (actually nucleotide-sugars, the derivative of nucleotides) is used as the key signal for activating and driving the cell cycle program, leading to cell division [33]. Hence, such cells use cell proliferation as a way to store unutilized energy. At the end of this process, the negatively charged DNA wraps around the positively charged histone-like proteins, which are together put into the daughter cell to maintain cellular electroneutrality.

To examine if Fenton reaction-affected cells may have evolved to “create” and utilize a similar program, we have conducted functional analyses of ALL the point mutations observed in cancer genomes in the 14 cancer types. To our great surprise, 40%–60% of all the mutations selected by each cancer type are related to cell polarity, while the other mutations are relevant to interactions with other cell types and driving the cell cycle. The cell polarity of an organism is the cellular infrastructure for supporting the accurate and efficient execution of the cellular functions encoded in the genome, which also defines what functions a cell can and cannot do [34]. Generally, cellular functions with directionalities such as transportation, localization, shape determination, and molecular assembly are polarity related. We have shown that the vast majority of cell polarity and cell cycle genes functional (unmutated and uninhibited) in cancer, in general, can be well mapped to the corresponding genes in one of the earliest (and simplest) multicellular organisms, which, like unicellular organisms, use the concentration of nucleotide-sugars to drive the cell cycle program (unpublished data). Based on this, we postulate that cancer cells have simplified their cellular system by mutating and repressing certain cell polarity genes to revive a lost capability; namely, nutrient concentrations drive cell cycle progression for survival!

This prediction is strongly supported by recent studies showing that oncogenes have generally evolved from microbial genes involved in sensing the cellular levels of nutrients, such as *RAS* for guanine nucleotide, *MTOR* for amino acid, and *GCK* for sugar [35]; and the vast majority of genes functional in cancers originate from unicellular organisms [36]. Furthermore, the majority of the known tumor suppressor genes are cell polarity genes, which has also been observed by other researchers [34]. For example, *TP53*, *NOTCH1*, *APC*, and *VHL*, widely known “tumor suppressor genes”, are all key polarity genes. Putting these together, cancer cells may have revived the original functions of some ancient nutrient-sensing genes and enabled such genes to drive cell cycle programs through mutating (and repressing) genes that have joined the human genome later and created more sophisticated functions along with the old nutrient-sensing and now oncogenes. Furthermore, the predominant class of the mutated genes are those providing the infrastructure in support of executing the human cell functions. Hence, we have a new and more general view of the functional roles played by the “oncogenes” and “tumor suppressor genes” in the bigger picture of cancer evolution.

Another most conserved RM is the over synthesis and deployment of sialic or poly-sialic acids, each producing numerous protons with a detailed number depending on where a poly-sialic acid is deployed. Sialic acids are each negatively charged and deployed on (cancer) cell surface. Our discovery is that the negative charge carried by each sialic acid will create and increase an electrostatic repulsion between neighboring cells as they continue to accumulate on the cell surface, leading to increased mechanical compression on the relevant cells and ultimately driving their migration [37], [38]. Interestingly, it has been observed since the 1960s that cancer cells over synthesize and deploy poly-sialic acids on the cell surface, which are suggested to be associated with cancer metastasis [39].

**Figure 1:**
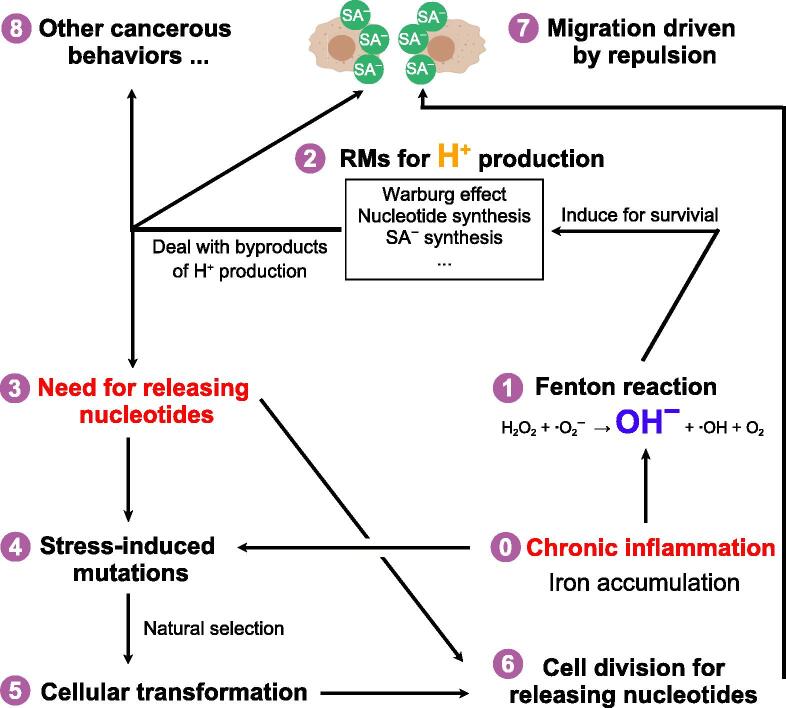
**Framework of cancerdevelopment** A diagram of how chronic inflammation coupled with iron overload may drive the formation and development of cancer, in which genetic mutations are predominantly used to simplify the human cells to a unicell-like organism. Numbers inside purple circles indicate the relative order of events. Cells in light brawn at the top of the diagram represent cancer cells, and green circles are for negatively charged SAs on the surface of cancer cells. SA, sialic acid; RM, reprogrammed metabolism.

Based on these and additional analyses, we postulate that the vast majority of the clinical phenotypes of cancer are the results of the RMs induced to keep cytosolic pH stable in cancer. Figure 1 shows a model for cancer development.

Overall, the model suggests that when a tissue is chronically inflamed at a level that leads to local iron accumulation, a Fenton reaction may take place. If this happens in the cytosol, the reaction will continuously put out OH^−^ at rates that can quickly overwhelm the pH buffer, hence driving the pH up. To survive, the affected cells activate a range of proton-producing metabolic processes, typically through piecing together segments of normal metabolic processes. One common process is the *de novo* synthesis of nucleotides. Our key prediction is that such cells utilize cell division to remove the nucleotides at rates comparable with the rates of OH^−^ production. To make this happen, cells must simplify their cell polarity to transform a human cell back to an ancestor unicell-like “organism”. Our general prediction is that most of the cancerous behaviors are the results of the RMs.

## Other contributing factors

**Figure 2:**
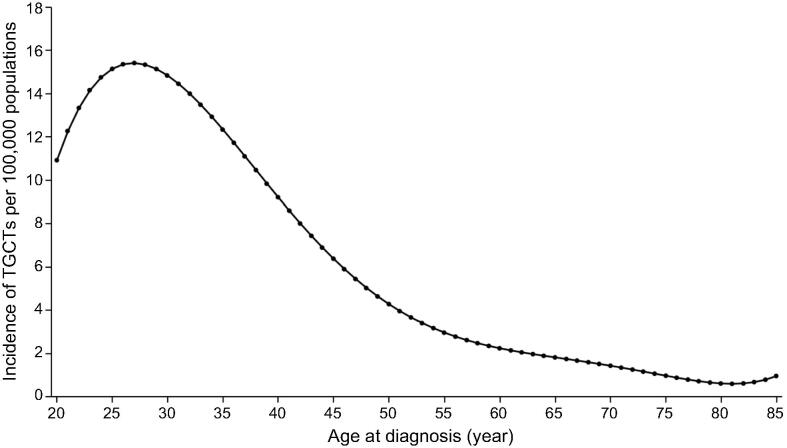
**Unimodal distribution of age-dependent occurrence of TGCTs** A unimodal distribution of age-dependent occurrence of TGCTs, where the x-axis is the age in years, and the y-axis is the cancer occurrence rate at a given age. The age-dependent cancer occurrence data were acquired from SEER reports. SEER, Surveillance, Epidemiology, and End Results; TGCT, testicular germ cell tumor.

While numerous characteristics of cancer can be readily or potentially explained using this model, some could not. For example, (1) why does the age-dependent occurrence rate of cancer generally follow a unimodal distribution *vs.* age, with the peak age possibly much lower than the life expectancy as shown in the Surveillance, Epidemiology, and End Results (SEER) reports [40] and visualized in Figure 2? And (2) why do certain organs have high cancer occurrence while others have low or virtually no occurrence [41]?

Aging has been considered a risk factor for cancer; however, for numerous cancer types, such as testicular and triple-negative breast cancers, their age-dependent occurrence rates strongly suggest that this view may not be supported by data. Instead, the shape of the curve suggests that there are other factors whose levels of contribution to cancer development go down with age, in addition to the cancer risk factors that increase with age [42]. We have recently developed a model suggesting that the age-dependent occurrence rate of cancer is the combined result of risk factors and the availability level of specific growth factors in circulation needed by each cancer type. When the required levels of growth factors are not available, the cells will die. Testicular cancer provides a good example here. Its occurrence rate peaks at a young age (Figure 2) and then drops with age, indicating that older patients have a reduced ability to develop cancer; instead, they suffer from testicular tissue loss, as reported in the medical literature [43], *i.e*., massive cell death as our model predicts. This suggests the possibility that cancer could be potentially treated by removing growth factors needed specifically by a specific cancer type or tissue.

Regarding the distinct cancer occurrence rates in different organs, the small intestine *vs.* colon serves as a good example, as the former has substantially lower cancer occurrence rates than the latter [40]. We have recently conducted a statistical analysis of gene expression data of numerous organs with a wide range of cancer occurrence rates in the Genotype-Tissue Expression (GTEx) database and found that the following characteristics of an organ may represent the key determinants of cancer occurrence rates in the organ: the intrinsic anti-inflammatory capability and the flexibility in maintaining homeostasis of various metal ions among a few others. This is derived based on a regression analysis of the known cancer occurrence rates of different organs against the average expression levels of the relevant cellular processes, which explains well the occurrence rates with high statistical significance (unpublished data).

In the bigger picture, the onset of cancer requires other stressors such as hypoxia and oxidative stress, which could be the results of chronic inflammation, persistent exposure to radiation, carcinogen, or certain microbes. We have previously predicted that persistent hypoxia may be an essential prerequisite for a tissue to become a soil where cancer can develop, namely a microenvironment rich in short hyaluronic acids [44], [45]. These hyaluronic acid fragments provide a wide range of signals for cell proliferation, cell survival, and angiogenesis, enabling highly abnormal cells with high rates of genomic mutations to avoid apoptosis and survive. Interestingly, the role played by hyaluronic acids decreases once the cells have completed their cancerous transformation and start again when cancer cells start to metastasize [44], [45]. Furthermore, we have also predicted that organisms capable of surviving persistent gaps between the ATP demand and supply under hypoxia may be an intrinsic characteristic needed for an organism to develop cancer, which explains why human, mouse, and rat can develop cancer but frog, turtle, and mole rat will not [44].

## Potential implications

The availability of large-scale omics data of cancer tissues has made it possible to develop testable models for cancer drivers and evolution. Our model represents one such effort. Two key distinctions of the model *vs.* others are: (1) it focuses on stresses and the adaptive steps made by the cells for survival, hence providing a natural framework for causal inference; and (2) stresses are studied at the levels of fundamental chemical homeostasis and physical balances, making the analyses at a more basic level compared to typical cancer studies.

The model provides a general framework for studies of functional relationships between cancer behaviors and molecular-level changes such as the RMs, enabling more focused and efficient ways to study cancer biology. We anticipate that the model, possibly with further development, is capable of explaining most, if not all, of the hallmarks of cancer. Since the model is generally applicable to possibly all cancers, we anticipate that it offers an effective framework for studying specific cancers by integrating the specifics of individual organs, allowing researchers to focus on major rather than minor issues in cancer biology. The current model has strong predictive power, as our previous work has suggested [9], that can be used to guide experimental designs for validation in a systematic manner. For example, we anticipate that the observed RMs being ultimately induced by the rising intracellular pH could be experimentally validated in the near future.

If the model proves largely correct, we anticipate that it will lead to fundamentally novel ways of diagnosing and treating cancers. For example, our model suggests that stopping key acidifiers, typically enzymes, should represent a novel way of stopping, hence killing the cancerous dividing cells.

## Competing interests

The authors have declared no competing interests.

## CRediT authorship contribution statement

**Renbo Tan:** Investigation, Data curation, Writing – original draft, Writing – review & editing. **Yi Zhou:** Formal analysis, Investigation, Writing – original draft, Writing – review & editing. **Zheng An:** Formal analysis, Visualization, Writing – review & editing. **Ying Xu:** Conceptualization, Writing – review & editing, Supervision. All authors have read and approved the final manuscript.

## Supplementary material

Supplementary data 1 **The list of reprogrammed metabolisms we have previously studied**
