# Supplementary material for: Cancer Is A Survival Process under Persistent Microenvironmental and Cellular Stresses
Source: Genomics Proteomics Bioinformatics. 2022 Jun 18;21(6):1260–5. doi: 10.1016/j.gpb.2022.03.002 (PMC11082257; doi:10.1016/j.gpb.2022.03.002)
Supplement: Supplementary data 1 — The list of reprogrammed metabolisms we have previously studied [file mmc1.docx]

**Table S1 The list of reprogrammed metabolisms we have previously studied**

| **Reprogrammed metabolic pathway** | **BioCyc pathway ID** | **PubMed ID** |
| --- | --- | --- |
| Acidifying transporters | - | 31932456 |
| Alkalizing transporters | - | 31932456 |
| Arginine transporter | - | 16670299 |
| ATP consumption | - | 31932456 |
| Beta oxidation | FAO-PWY | 12535077 |
| Ceramide synthesis | PWY3DJ-12, PWY3DJ-11470 | 9312549, 15258913 |
| Choline production and metabolism | PWY-7782 | 19906248 |
| Chondroitin sulfate synthesis | PWY-6567 | 12512856 |
| Circadian rhythm | - | 25961153 |
| Fatty acid synthesis | PWY-5966-1 | 27658529 |
| Fatty acid transporter | - | 31932456 |
| Gluconeogenesis | PWY66-399 | 31019893 |
| Glutaminolysis | GLUTAMINDEG-PWY, PWY66-398, 1.1.1.39-RXN | 28393116 |
| Heparan sulfate synthesis | PWY-6558 | 12512855 |
| Hyaluronic acid synthesis | UDPNACETYLGALSYN-PWY, PWY-7346 | 16408321 |
| Hydroxylation enzymes | - | 31932456 |
| Keratan sulfate synthesis | KERATAN-SULFOTRANSFERASE-RXN | 6592165 |
| Lysine degradation | LYSINE-DEG1-PWY | 1909881 |
| Methylation | - | 31932456 |
| Mevalonate metabolism | PWY-922 | 9270019 |
| NAD^+^ synthesis and metabolism | - | 31932456 |
| N-linked glycosylation complex phase | PWY-7426 | 24178944 |
| N-linked glycosylation initial phase | MANNOSYL-CHITO-DOLICHOL-BIOSYNTHESIS | 9878760 |
| O-linked glycosylation | PWY-7433 | 27686492 |
| Phospholipid degradation | PHOSPHOLIPASE-A2-RXN, PWY66-375 | 6252969, 17623009 |
| Phosphatidic acid (PA) synthesis | PWY-5269 | 10777514 |
| Phosphatidylcholine (PC) synthesis | PWY3O-450 | 18204095 |
| Phosphatidylethanolamine (PE) synthesis | PWY4FS-6 | 23124206 |
| Phosphatidylinositol (PI) synthesis | PWY-6352 | 8086005 |
| Phosphatidylserine (PS) synthesis | PWY-7501 | 19014349 |
| Proline synthesis | PROSYN-PWY | 2722838 |
| Phosphorylation/dephosphorylation | - | 31932456 |
| Purine dRN *de novo* synthesis | Purine-Deoxyribonuc-De-Novo-Biosynthesis | 31932456 |
| Purine dRN salvage | PWY-7224 | 7494863 |
| Purine RN *de novo* synthesis | Purine-Ribonuc-De-Novo-Biosynthesis | 31932456 |
| Purine RN degradation | PWY0-1296 | 12180982 |
| Pyrimidine degradation | PWY0-1295 | 10354618 |
| Pyrimidine dRN *de novo* synthesis | PWY-7184 | 12369616 |
| Pyrimidine dRN salvage | PWY-7199 | 7494863 |
| Pyrimidine RN *de novo* synthesis | PWY-7176 | 12369616 |
| Pyrimidine RN salvage | PWY-7193 | 16769123 |
| Retinol metabolism | PWY-6875 | 18569334 |
| Retinol synthesis | PWY-6857 | 18805086 |
| Serine synthesis | SERSYN-PWY | 12633500 |
| SA synthesis | PWY-6138 | 15888312 |
| Triglyceride degradation | LIPAS-PWY | 16916618 |
| Triglyceride synthesis | TRIGLSYN-PWY | 19355855 |
| Tryptophan degradation | TRYPTOPHAN-DEGRADATION-1 | 22084578 |
| Warburg effect | - | 26113084 |
